# Recent Advances in Recovery of Lycopene from Tomato Waste: A Potent Antioxidant with Endless Benefits

**DOI:** 10.3390/molecules26154495

**Published:** 2021-07-26

**Authors:** Valentina Noemi Madia, Daniela De Vita, Davide Ialongo, Valeria Tudino, Alessandro De Leo, Luigi Scipione, Roberto Di Santo, Roberta Costi, Antonella Messore

**Affiliations:** 1 Istituto Pasteur-Fondazione Cenci Bolognetti, Dipartimento di Chimica e Tecnologie del Farmaco, “Sapienza” Università di Roma, p.le Aldo Moro 5, I-00185 Rome, Italy; valentinanoemi.madia@gmail.com (V.N.M.); davide.ialongo@uniroma1.it (D.I.); valeria.tudino@uniroma1.it (V.T.); alessandro.deleo@uniroma1.it (A.D.L.); luigi.scipione@uniroma1.it (L.S.); roberto.disanto@uniroma1.it (R.D.S.); antonella.messore@uniroma1.it (A.M.); 2 Department of Environmental Biology, “Sapienza” University of Rome, p.le Aldo Moro 5, I-00185 Rome, Italy; daniela.devita@uniroma1.it

**Keywords:** lycopene, carotenoids, food waste, nutraceuticals, supercritical fluid extraction, pulsed electric fields treatment, enzyme-assisted extraction, supercritical fluid extraction, ultrasonic-assisted extraction, microwave-assisted extraction

## Abstract

Growing attention to environmental protection leads food industries to adopt a model of “circular economy” applying safe and sustainable technologies to recover, recycle and valorize by-products. Therefore, by-products become raw material for other industries. Tomato processing industry produces significant amounts of by-products, consisting of skins and seeds. Tomato skin is very rich in lycopene, and from its seeds, high nutritional oil can be extracted. Alternative use of the two fractions not only could cut disposal costs but also allow one to extract bioactive compounds and an oil with a high nutritional value. This review focused on the recent advance in extraction of lycopene, whose beneficial effects on health are widely recognized.

## 1. Introduction

One of the major drawbacks in the food life cycle is food waste, inevitably produced during all the stages of the manufacturing process. These phases include agricultural production, industrial processing and distribution. With the aim to reduce the impact on the environment and, at the same time, give wastes a second life, in the last decade, there has been growing interest in reusing and upgrading food by-products from the processing industry. Indeed, food waste and by-products are a good source of high-value components, such as proteins, polysaccharides, fibers, flavor compounds and phytochemicals, as nutritionally and pharmacologically functional ingredients [1].

To preserve the resources and minimize the high costs of waste disposal, the new trend consists in the waste recovery processing residues as potential sources of compounds with active properties (nutraceuticals and functional ingredients) [2].

Nutraceuticals and functional ingredients present in agro-industrial waste can be recovered using various techniques that have been successfully applied for recovery and reuse of waste from industrial tomato processing. Tomato is the fruit berry of *Solanum lycopersicum* L., belonging to Solanaceae family. It is a native species to South America, domesticated in Mexico and introduced as an ornamental plant into Europe where it was considered poisonous before it was considered useful as a vegetable [3]. In 2019, Italy was the main European producer followed by Spain and Portugal: more than 10 million tons of tomatoes produced in Europe were processed to produce peeled tomatoes and juices, among others [4]. Tomato processing generates high amounts of by-products that represent an environmental inconvenience for producers but at the same time a source of bioactive compounds [5]. According to the common processing, industries produce as tomato by-products (TBPs) peel, seeds or pomace which consists mainly of peel, seeds and a small amount of pulp and represents up to 5% of the whole tomato [6]. TBP is a source of valuable compounds: minerals, vitamins dietary fibers, proteins, polyphenols and carotenoids [7].

The latter are yellow, orange and red pigments present in many plants. From a chemical point of view, these compounds are water-insoluble, with a conjugated polyene chain. Most of the carotenoids are tetraterpenes, with a 40-carbon skeleton; carotenoids with a 45- or 50-carbon skeleton, the so-called higher carotenoids, are known [8]. Moreover, carotenoids with fewer than 40 carbons (apocarotenoids) are present in nature as a result of a degradation of C40 carotenoid precursors, such as lycopene and β-carotene [9]. Carotenoids can be divided into two groups: carotenes and xanthophylls. Carotenes, including β-carotene and lycopene, are hydrocarbons, whilst xanthophylls contain oxygen atoms and in some cases are present as fatty acid esters, glycosides, sulfates and protein complexes [8]. One of the main carotenoids recovered from tomato waste is lycopene, besides β-carotene and lutein [5]. Lycopene is an acyclic carotene, with 11 conjugated double bonds. Lycopene is a phytonutrient playing an important role in human health. Indeed, it has long been known for its several biological activities such as antioxidant [10], anti-inflammation [11], hypoglycemic [12,13], photoprotection [14], anti-angiogenesis [15], anti-parasitic [16,17,18,19], antiviral [20] and others. Several studies have also indicated the effect of dietary lycopene in reducing the risk of chronic diseases such as coronary heart disease [21], being also useful in reducing the oxidative stress in human immunodeficiency virus (HIV)-infected patients under antiretroviral therapy that have less total antioxidant capacity [22,23,24]. Moreover, lycopene ameliorates skin and bone diseases and hepatic, neural and reproductive disorders with a mechanism of action that has been investigated [10,25].

**Figure 1:**
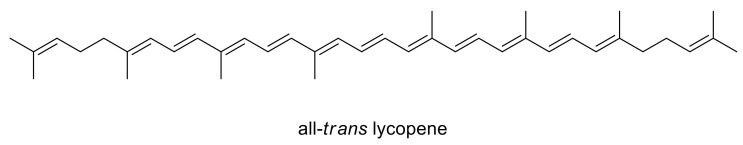
Structure of all-*trans* lycopene.

All-*trans* lycopene (Figure 1), the most thermodynamically stable form [26], is the predominant species in tomato; however, the double bonds can undergo isomerization producing mono- and poly-*cis* isomers by effect of light and/or heat [27]. Lycopene, like other carotenoids such as β-carotene, is sensitive to light, heat and oxygen, and it may easily undergo (*trans–cis*) isomerization, thus resulting in an array of mono- or poly-cis isomers [28]. Several studies have shown that heat treatments, longer than 1 h, favored the *trans–cis* isomeric conversion of lycopene while light irradiation induced *cis*-isomer degradation over time in tomato products [29,30]. Worth of note is that lycopene-based food products are very dynamic systems: indeed, during food processing, the proportion of *cis*-isomers increases, and retro-isomerization during storage of processed foods happens [31] because *cis*-isomers are more unstable when compared to *trans*-isomers. Actually, even if lycopene prevalently exists in nature in the all-*trans* form, it has been suggested that the bioavailability of *cis*-lycopene in foods is higher than that of all-*trans* lycopene: *cis*-isomers are better absorbed due to the greater solubility in mixed micelles which favor intestinal absorption; moreover, the content of *cis*-isomers of lycopene in body tissues is believed to also depend on transport, degradation and heat-induced isomerization within the mammalian tissue [32] promoting in vivo *trans*-to-*cis* lycopene isomerization [28,33]. Given the predominance of the all-*trans* form (that is also the most stable under a thermodynamic point of view), the need to avoid the *trans*-to-*cis* isomerization for incorporating lycopene into nutraceuticals or functional foods is evident.

The composition and structure of the food matrix also influence the bioavailability of lycopene: indeed, cooking or shredding can increase bioavailability due to physical destruction or a softening of the plant cell membranes and through the breakdown of lycopene-protein complexes [34].

To date, the demand for functional food, supplements and cosmetics containing lycopene is rapidly growing. In order to improve the recovery of lycopene from natural sources, especially from solid waste resulting from the industrial processing of tomato, several approaches are used.

Organic solvents are used in traditional processes (i.e., fractional distillation, steam distillation, solvent extraction) to extract bio-compounds, such as lycopene, from plant materials. However, the use of such solvents poses several drawbacks for both human health and environment safety. Indeed, organic solvents not only create environmentally hazardous problems, but also their residues remaining in the final products become a major safety concern. The current concern for safety in food products has raised interest in “green” extraction techniques as alternative methods to the conventional extraction processes. This review aims at summarizing the most recent advances in conventional and novel techniques suitable for extracting lycopene from TBPs (Table 1) by providing an updated account of the main progress in this field of research. These data could hopefully improve the awareness in reducing food waste as much as possible and increase the reuse of components from processing industry by-products.

## 2. Extraction of Lycopene from TBPs

Classical Organic Solvent Extraction Technique (COSE)

The amount of lycopene recovered from tomato waste strictly depends on starting material and extraction methods. Because of its non-polar nature, lycopene can be extracted from suitable organic solvents [36,37]. In addition to the choice of the appropriate solvent, it is necessary to optimize several parameters including ratio solvent/matrix, temperature and extraction time in order to increase the recovery of the target compounds. The most common solvent techniques for the extraction of lycopene for TBPs are extraction by Soxhlet apparatus or mechanical stirring. The former allows a higher extraction yield; however, long time and high temperature applied during Soxhlet extraction can degrade heat-sensitive compounds [38]. Mixture design procedure can be used to formulate solvent mixtures to recover lycopene. A ternary system (*n*-hexane-ethanol-acetone) was investigated resulting in a lycopene extraction yield higher than 95% and providing a tomato oleoresin with high lycopene content [39]. The main drawback of the conventional solvent-based extraction procedures is the toxicity of traces of solvents that can remain causing long-term effects including leukemia [40], renal injury [41] and neurotoxicity [42]. Moreover, an increase in production cost is expected in the evaporation of the solvent and in the purification step. Despite the efforts in optimizing the solvent extraction process over the last decade, this method has a high environmental impact, and therefore new and more eco-sustainable techniques are investigated.

## 3. Pretreatments of TBPs Before Extraction

### 3.1. Pulsed Electric Fields (PEF) Treatment

Pulsed electric fields (PEF) treatment is a method that uses a potential difference between two electrodes among which biological materials are placed. The application of electric field (0.5–80 kV/cm) pulses on plant matrices induces electropermeabilization allowing a higher extraction yield of plant metabolites [43]. At first, the cell disintegration index must be used to find the conditions where the degree of cell membrane permeabilization is the highest. After that, a PEF pre-treatment with moderate electric field intensity and low energy input increases the permeabilization of membranes of plant cells, enabling high recovery yields of intracellular compounds before solvent extraction [44]. PEF has been used successfully for the extraction of tomato pulp which leads to samples with a higher concentration of lycopene than the untreated samples [45] and subsequently applied to the recovery of lycopene from tomato waste. In a study by Pataro et al. [46], a PEF processing was performed on tomato peel before being subjected to extraction with acetone at different temperatures up to 50 °C. Compared to untreated samples, the optimal energy input of 5 KJ/Kg at the field strength of 5 kV/cm enhanced the total carotenoids content and the ferric-reducing antioxidant power of the extract. As confirmed by high-performance liquid chromatography (HPLC) analysis, no isomerization or degradation of carotenoids occurred [46]. This method has been applied efficiently before peel extraction with either acetone or ethyl lactate [47] or on juicing residues before acetone extraction [48], obtaining higher lycopene yield with acetone which showed better capability to penetrate the plant cells of peel tissue and to solubilize a greater amount of intracellular lipophilic compounds.

### 3.2. Enzyme-Assisted Extraction (EAE)

This eco-sustainable technique involves the use of enzymes such as cellulase, pectinase and hemicellulase that catalyze the hydrolytic cleavage of structural components of the cell wall of the waste product; consequently, an increase in permeability allows one to reach high extraction yields. In addition, EAE of compounds from natural matrices leads to a reduction in extraction time and in using an organic solvent. Nevertheless, EAE has some limitations that include enzyme cost for processing high amounts of matrices; moreover, the enzymes currently available cannot completely hydrolyze cell walls [49]. In this context, the effects of cellulase and pectinase before the extraction of lycopene by petroleum ether and acetone (1:1 *v*/*v*) were investigated [50] showing the pectinase more effective than cellulase in obtaining lycopene from tomato peels. A similar result was reported by Ranveer et al. 2013 [51] who found higher lycopene recovery for tomato peel treated with 2% pectinase and extracted with hexane:acetone:ethanol 50:25:25 *v*/*v*. This enzymatic pretreatment was then optimized (2% pectinase, pH 5.5, 4 h incubation, 45 °C) [52]. Furthermore, the recovery of carotenoids by using a mixture of hydrolytic enzymes was found to be a good approach [53]. The recovery of lycopene from tomato waste (peel and seeds) was extensively investigated [54] by using a pectinolytic enzyme (Pectinex Ultra SP-L), a cellulolytic enzyme (Celluclast) and a combination of carbohydrases (including arabanase, cellulase, β-glucanase, hemicellulase and xylanase, named Viscozyme L) alone and in combination. A mixture of Celluclast and Pectinex (T = 40 °C, enzymatic reaction time = 5 h, enzyme:substrate ratio = 0.2 mL/g, enzyme:enzyme ratio = 1) followed by ethyl acetate extraction (solvent:substrate ratio = 5 mL/g, extraction time = 1 h) showed the highest lycopene recovery. The study conducted by Lavecchia and Zuorro [55] investigated the pretreatment of tomato peel with four food-grade enzyme preparations (Citrozym CEO and Ultra L, Peclyve EP and LI) with pectinolytic, cellulolytic and hemicellulolytic activities followed by extraction with hexane, finding that the enzyme preparation Peclyve LI enhances more lycopene extraction from peels. After that, the effect of solvent extraction on yields was investigated using hexane, ethyl acetate or a ternary mixture (hexane:acetone:ethanol 50:25:25 *v*/*v*/*v*) at different incubation times (0.5–15 h). After the pretreatment with the enzymes, the extraction yield of lycopene was 3- to 25-fold higher than the untreated matrix, with the highest yield obtained with the ternary mixture after 1 h of enzymatic treatment since longer reaction time leads probably to oxidation of lycopene. More recently, the same authors [56] investigated the optimization of the process by response surface methodology (RSM), based on statistical and mathematical techniques. The factors investigated included extraction temperature, extraction time and enzyme load with a mixture of Peclyve PR and Cellulyve 50 LC in equal volumes to catalyze the enzymatic reaction, finding the following optimal extraction conditions: T = 30 °C, extraction time = 3.18 h and enzyme load = 0.16 kg/kg. A similar approach was followed by Lombardelli et al. [57] in order to recover carotenoids from unsold tomatoes: a mixture of polygalacturonase, pectin lyase, cellulase and xylanase was investigated, while the optimal conditions (50 °C, pH = 5.5) by using an enzymatic mixture with a total dosage of 25 U/g for 180 min were identified by the RSM analysis. Interestingly, the extraction was performed by an environmentally friendly condition, as proposed by Cuccolini et al. [58]. The method involves first treatment with NaOH solution which allows one to open the tomato peel cell wall, dissolving the layer that cements the cells, and the subsequent enzymatic hydrolysis by cellulase and pectinase allowing to isolate the carotenoid-accumulating plastids named chromoplasts. Lycopene is stored inside the chromoplasts incorporated into lipoproteins that confer its stability. At this point, a protease eliminates proteins bound to the chromoplasts, providing a final product lycopene-rich with a 30-fold increase in lycopene contents compared to untreated peels.

## 4. Supercritical Fluid Extraction (SFE) of TBPs

A promising eco-friendly method is supercritical fluid extraction (SFE) that uses supercritical CO_2_ (scCO_2_) “green technology” for its advantages. Indeed, it is harmless, environmentally safe and highly selective because of low viscosity, high diffusivity and liquid-like density [59]. Furthermore, it is quickly accessible and simply removed from products, and, also, critical CO_2_ temperature is close to room one, and therefore it is also suitable for thermolabile substances [60]. Due to its non-polar nature, CO_2_ has been used successfully for the extraction of non-polar compounds from plants [61,62]. SFE demonstrated to be a proper method for the extraction of lycopene without organic solvent residues, thus overcoming the main issues in extracting lycopene: its solubility and stability. Indeed, lycopene is water-insoluble but soluble in highly toxic organic solvents and decomposes easily over time. There are numerous reports describing the successful extraction of lycopene from tomato materials by SFE plus several studies reporting the effects of different independent variables, such as pressure, temperature, flow rates and co-solvent or modifier concentrations. The choice of the SFE operating conditions is necessary for reaching the best possible extraction efficiency, that is, the maximum amount of material that can be extracted at a certain temperature, pressure and flow rate. Indeed, the optimization of such parameters is needed to achieve a high recovery of lycopene. Kehili et al. extracted lycopene and β-carotene from TBP peels with scCO_2_ at 50–80 °C, 300–500 bar and flow rates of 3–6 g CO_2_/min for 105 min on ground tomato peels of 300 µm particle size [63]. The relative extraction yields ranged from 32.02% to 60.85% for lycopene and from 28.38% to 58.8% for β-carotene, considering as maximum extractable amounts 1198 ± 72 mg/kg and 27.94 ± 0.06 mg/kg, on a dry basis, of lycopene and β-carotene, respectively, obtained with Soxhlet extraction with *n*-hexane for 12 h. In particular, the maximum lycopene (60.85%, 728.98 ± 31 mg/kg) and β-carotene (58.8%, 16.43 ± 0.84 mg/kg) recoveries were obtained under 400 bar, 80 °C and 4 g CO_2_/min. Furthermore, a comparison between SFE and conventional maceration extraction was made, starting from the same TBP peels as for SFE. This method produced 608.94 ± 10.05 mg/kg, 320.35 ± 3.4 mg/kg and 284.53 ± 7.0 mg/kg using *n*-hexane, ethyl acetate and ethanol, respectively. The *n*-hexane was the most convenient solvent, 50.83%, while ethyl acetate and ethanol led to lower yields, 26.74% and 23.75%, respectively. Therefore, the authors concluded that scCO_2_ provided higher lycopene recovery with respect to solvent extraction, despite the limited solubility of lycopene in supercritical fluids. Rozzi et al. [64] determined the temperature, pressure, flow rate and CO_2_ volume effects on SFE of lycopene from a by-product of tomato processing. The parameters were evaluated on the recovery in tomato seeds and skins (51.6% dry matter). It was proven that both temperature and pressure influenced the extraction of lycopene and that an optimum temperature and pressure combination resulted in extracting 61.0% of the lycopene present in the sample. The authors hypothesized that this optimum could be ascribed to a decrease in diffusivity of CO_2_ as its density increases which could hinder the CO_2_ capability of diffusing throughout the sample, thus dissolving more solute. Another hypothesis was that the pressure increase could lead to a compaction of the sample, similarly hindering the ability of CO_2_ to diffuse. SFEs were carried out with scCO_2_ at seven temperatures (32–86 °C) and six pressures (13.78–48.26 MPa). The effect of scCO_2_ flow rate and volume was also evaluated. Results indicate that the amount of extracted lycopene increased with high temperature and pressure until a limit at 86 °C and 34.47 MPa, after which it decreased. Furthermore, the optimum conditions to extract lycopene from 3 g of raw material were defined using a flow rate of 2.5 mL/min and 500 mL of CO_2_ (indeed, a higher amount of CO_2_ led to a lycopene amount plateau). These conditions granted 61.0% of lycopene extraction (7.19 µg lycopene/g). Notably, the initial lycopene content of the tomato seeds and skins was 11.8 µg of lycopene per gram of raw material (wet weight) or 24.5 µg of lycopene per gram of dry material (dry weight) as determined by chloroform extraction. The influence of temperature as a parameter for reaching an optimum in SFE of lycopene was also confirmed by Pellicano and coworkers [60]. Even so, at the temperature of 80 °C, the authors observed a decrease in the lycopene solubility in scCO_2_, probably due to its thermal degradation. For this reason, they decided to operate at 60 °C. Indeed, they observed a slight increase in recovering lycopene when the temperature rose from 40 to 60 °C and achieved a plateau before reaching 80 °C. Pressure effects on lycopene extraction were similar to temperature ones. Indeed, the lycopene recovery increased with the pressure, reaching a plateau (corresponding to the maximum yield of extraction of 4.37%, that is, 0.79 g, from about 18 g of dehydrated tomato seeds and skins) at 550 bar after 80 min. The authors claimed that lycopene recovery is mainly dependent on the interaction between pressure and temperature. Indeed, the pressure increase leads to higher CO_2_ density which in turn determines an increase in the solvating power of the supercritical fluid (with a consequent greater lycopene extraction). This effect was also observed by Topal et al. who noticed that higher pressure is responsible for quantitative recoveries and stronger interactions between the fluid and the matrix [65]. On the other hand, a higher temperature means a density decrease (and therefore a decrease in the solvent power) while keeping constant the pressure parameter. Even so, one must account for two factors: (a) the density decrease becomes smaller at high pressures, and (b) the rise in temperature means also an increase in solubility (due to the sublimation temperature increase in the solute) that opposes the density decrease. Therefore, lycopene solubility in CO_2_ increases when temperature and pressure parameters are both increased. By exceeding in elevating the temperature, degradation through isomerization and auto-oxidation of lycopene was often encountered, as observed by Mayeaux et al., especially during long heating times [66]. The effects of temperature and pressure on SFE were also investigated by Vàgi and colleagues who analyzed also the influence of air-dried and deep-frozen storages on lycopene recovery [67]. The authors obtained the highest concentration of carotenoids with 90.1% of lycopene at 460 bar and 80 °C (no degradation of the biologically active compounds happened), observing that the extraction yields were higher of the sample stored at deep-frozen state for 1 year than that stored at room temperature for the same time. In particular, the amount of carotenoids in the deep-frozen stored sample was 10 times higher than that present in the air-dried stored sample, with lycopene conversed in a higher amount too. Interestingly, Zhang and colleagues studied the effect of the operating parameters on lycopene scCO_2_ extraction from freeze-dried tomato peels and seeds [68]. In particular, the authors investigated the variation of extraction yield and lycopene content at different temperatures (40 °C, 50 °C and 60 °C), pressures (35 MPa, 40 MPa and 45 MPa) and particle size (three-degree index). The highest lycopene concentration was obtained at 40 MPa, 60 °C (32.52 g/100 g dry material) and with the mean particle sizes of tomatoes peels and seeds powder of 0.30 mm, while the extract rich in β-carotene was obtained at below 30 MPa. Another important parameter is the flow rate of scCO_2_. It influences the extraction efficiency by controlling the amount of solvent (that is, CO_2_) to be used and the extraction time. The higher the flow rate used, the faster extractions and the higher recoveries are obtained since it can overcome the interface resistance for lycopene transport from tomato skin to CO_2_. However, exceeding flow rates produces undesirable results due to decreased contact times between solvent and solute [65]. The extraction time is another important factor influencing the yield of the process. For the extraction of lycopene, Baysal and coworkers [69] observed that the highest yield was obtained at an extraction time of 2 h, compared to 1 or 3 h. In the former case, the extraction time was not sufficient to dissolve the solute in the solvent, while over 3 h the occurrence of degradation increased. Remarkably, Huang et al. reported the effects of temperature, pressure and extraction time on lycopene yield from tomato pomace utilizing ethanol-modified scCO_2_ [70]. They observed that not just pressure and temperature influenced lycopene yields, but it was more precisely the interaction between pressure, temperature and extraction time. A polynomial regression model was suggested that allowed identifying the optimum conditions at 57 °C, 40 MPa and 1.8 h. These conditions led to a lycopene yield of 28.64 mg from 100 g dried tomato pomace (recovery = 93%). An important role in scCO_2_ extraction of lycopene is also played by co-solvents or modifiers, employed to enhance extraction efficiency and cost-effectiveness. Modifiers are like co-solvents in that they aid in the extraction, but modifiers are added directly to the sample before extraction, instead of with the solvent (like co-solvents) [64]. Numerous chemical modifiers (such as water, ethanol and methylene chloride) were tested to enhance the extraction process. Co-solvents or modifiers added in small amounts (1 to 5% mol) can modify the overall extraction fluid characteristics in terms of polarity, solvent strength and specific interactions. As a result of these modifications, a significant alteration of density and compressibility of the supercritical fluid happens. The addition of small amounts of water, vegetable oil or ethanol can significantly enhance the extraction of non-polar compounds such as lycopene. Shi et al. reported the use of ethanol as a modifier [71], observing that the ethanol, diluting the extract, reduced the viscosity and thus enhanced the extraction flow. The increase in recovery showed a linear correlation with that of the ethanol concentration at 45 °C when it was increased from 5 to 10% (lycopene recovery increase of 11%). A further increase from 10 to 15% led to a lower increase in lycopene recovery (6%). These results highlight the importance of selecting the proper concentration of the modifier to improve extraction efficiency. Another advantage of adding co-solvents or modifiers is the ability to swell the matrix, favoring the penetration of the CO_2_. The lycopene recovery increased by increasing the water concentration in tomato skin materials, even if an excess of the water content (higher than 18%) causes mechanical difficulties due to a material transport impairment [72]. In this regard, the moisture content must be considered for SFE. Given the lipophilic properties of lycopene, edible oils as alternative modifiers were considered to improve SFE. It was also observed that the presence of co-solvents has a beneficial role in pigment stability [73]. Moreover, one might keep in mind that such modifiers have the advantage of being edible, and thus they do not need to be subsequently separated from the product. Several vegetable oils were studied (almond oil, peanuts, hazelnuts and sunflower seeds) by Margotta et al., but only in the case of hazelnut oil (cheaper and with a low acidity preventing the lycopene degradation during extraction) higher extraction yields were achieved [73]. As mentioned above, given that a higher quantity of oil provides a more diluted extract, the authors decided to choose an average value of 10%. They observed that this oil allowed avoiding the degradation of lycopene, keeping the pigment stable over time. However, the use of organic solvents has two drawbacks: (a) it is not specific for lycopene, thus extracting many other pigments (carotenes, xanthophylls) and so causing a long and expensive purification process of lycopene; and (b) the raw material used becomes a special waste that will have to be incinerated with a considerable cost increase. Differently, Perretti et al. studied the SFE of lycopene from dried tomato pomace using sunflower oil and ethanol [74]. After the supercritical fluid fractionation, the authors collected four fractions (at 30, 60, 120 and after 120 min) and analyzed the lycopene concentration under different pressures (10 and 30 MPa) and flow rates (5 and 15 kg/h). The best recoveries were obtained in the 30 min fraction operating at 10 MPa and flow rate of 5 kg/h and in the 120 min fraction operating at 30 MPa and 15 kg/h. Moreover, by comparing the lycopene concentrations of the fractions obtained at 30 and at 60 min in the same operating conditions, the higher content of lycopene of the 30 min fraction confirmed the positive role of ethanol as a co-carrier, providing the higher solubility of lycopene in the presence of this solvent in the column. Interestingly, Machmudah et al. described the use of tomato seed oil as an alternative to organic solvents to increase the solvating power of scCO_2_ in order to reduce the drawbacks of organic solvents [75]. Thus, they studied the effects of temperature, pressure and flow rate on lycopene and β-carotene recovery using tomato seed oil as a co-solvent as well as the composition of oil from the tomato seed. The lycopene and β-carotene recovery was considerably enhanced with tomato seed oil as a co-solvent and, after 120 min of extraction, was basically constant. In addition, an increase in the recovery of lycopene, β-carotene and tomato seed oil was observed by increasing pressure but not the temperature. Indeed, the β-carotene recovery slightly decreased with higher temperature, probably due to both a higher degradation of β-carotene and to a predominating effect of the density decrease than the solute vapor pressure increase (by increasing the temperature). Conversely, the lycopene recovery slightly increased by increasing temperature due to an increased vapor pressure of the lycopene that causes an increased lycopene solubility. Concerning the tomato seed oil extraction, a temperature increase did not significantly affect the recovery. Furthermore, the CO_2_ flow rate did not appreciably influence the lycopene, β-carotene or seed oil recovery. The authors argued that the solubility of the solute in scCO_2_ limited the extraction. The tomato peel/seed ratio was also important on lycopene and β-carotene recovery because an increase in recovery was observed by increasing the seed amount. Still, this was true up to a maximum peel/seed ratio because further increase led to a decrease in the recovery. The authors hypothesized that this increase could cause a higher extraction of seed oil that may hinder CO_2_ transport into the solid matrix. A fundamental aspect that needs to be considered is the degree of degradation via isomerization and oxidation of lycopene and β-carotene. Therefore, several attempts have been described in the literature to study the possibility of obtaining the most stable isomer (all*-trans* form) with SFE, avoiding the oxidative degradation and the formation of artefactual isomers during the process. Gòmez-Prieto et al. reported a method of SFE that allowed minimizing the *trans*-to-*cis* isomerization of lycopene [28] without using co-solvents. Given this as the main purpose of their work, the authors maintained an extraction temperature as low as possible, optimizing the lycopene solubility by setting an adequate pressure parameter (and the density of CO_2_ consequently). In particular, they noticed that keeping the temperature at 40 °C and varying the pressure between 77 and 281 bar, a negative or non-significant effect of the temperature is obtained, probably due to a balance between the supercritical fluid density and the solute vapor pressure. Moreover, the extraction times were set as low as possible (30 min) in order to further reduce the possibility of isomerization. The authors observed that the amount of *trans*-lycopene rose (and the *cis* form decreased) as the pressure increased due to the corresponding increase in CO_2_ density. In particular, they obtained an extract with 88% all*-trans* lycopene and 12% *cis*-lycopene working at 281 bar (providing a density of 0.90 g/mL), ascribing the presence of a *cis*-isomer to the solubility differences of *trans* and *cis* forms in CO_2_ rather than their trend to undergo isomerization. Further studies reported that no dramatic effect on the isomerization degree happens at temperatures below 75 °C as reported by Mayer-Miebach et al. [76] or below 70 °C (as described by Yi et al.) [72]. Indeed, the ratio all*-trans*:*cis*-lycopene showed the major influence on the lycopene isomer composition at temperatures above 70 °C, with variations from 1.70 to 1.62 at extraction temperatures between 40 °C and 70 °C and from 1.62 to 1.32 at temperatures between 70 °C and 100 °C. These modifications in the all*-trans*:*cis*-lycopene ratio indicated the generation of the *cis*-isomers. Interestingly, Shi et al. described an increase in the proportions of *cis*-isomers using olive oil as a modifier rather than ethanol or water. The authors explained this behavior by that *cis*-isomers are less prone to crystallization and more soluble in lipophilic solvents such as oil.

## 5. Ultrasonic-Assisted Extraction

The use of ultrasounds has been successful in various food industry processes such as emulsion, crystallization, filtration, separation, defoaming, extrusion, fermentation and microbial inhibition [77,78,79]. The sound waves produced by an ultrasound probe at frequencies higher than human hearing cause a mechanical impact, allowing greater penetration of the solvent into the plant body, known as the “sponge effect”. In addition, high-energy cavitation bubbles containing solvent vapor are created. These bubbles, imploding near the cell walls, cause very high local temperatures and an increase in pressure that cause the destruction of the cell walls. The combination of these effects intensifies the penetration of the solvent and satisfies a sufficient mixing to extract high quantities of active components. In addition to facilitating extraction, ultrasound, however, can also produce highly reactive free radicals in solutions [80,81,82].

In the case of lycopene, some recent studies show that ultrasonic extraction accelerates the extraction rate and increases the yield by about 10% [83,84,85].

In a 2014 study by Yilmaz et al. [86], the difference in extraction of lycopene and β-carotene from industrial tomato waste was evaluated between the classical organic solvent extraction technique (COSE) and the ultrasound technique. With normal organic solvents, yields increase over time. In detail, there is growth for extraction periods of 10 min and 20 min while for longer periods of 30 and 40 min, for example, the yields are similar for both lycopene and β-carotene. This can be explained by the decrease in the driving force of the osmotic equilibrium since the diffusion of carotenoids from the material to the solution in COSE occurs slowly so that the osmotic pressure between the inside and outside of the cell easily reaches equilibrium [87,88]. Using ultrasound, on the other hand, it was observed that the extraction yield increased exponentially already at 2 min, then increased more gradually at 10 min, to finally become constant during the extraction. The initial large increase in the extraction rate may be due to the large concentration gradient of β-carotene and lycopene between the pure extractor solvent and the material to be extracted. Subsequently, the concentration gradient decreases as the extraction proceeds and the extraction becomes more and more difficult thanks to the internal position of the cells. Similar results were observed for the extraction of all-*trans* lycopene from red grapefruit and lycopene from tomato processing waste [88,89]. In previous studies, comparable results were reported for the ultrasonic extraction of all-*trans* lycopene and time was found to be the most important factor affecting the extraction yield. Most *trans*-lycopene could be extracted as early as the first third of the total extraction period. At this point, phenomena of degradation and the isomerization of lycopene would take place, leading, in fact, to a reduction in the quantity of substance. This could be attributed to the side effect of sonication called ultrasonic degradation [88,89,90,91,92]. With a significant increase in power from 50 W to 65 W, then, the value of lycopene increases significantly, while the increase in the value of β-carotene between 50 W and 65 W is not significant. Instead, the application of 90 W has a significant effect on both lycopene and β-carotene. Cavitation and thermal effects play an equally important role. With an increase in potency, more energy is transferred for cavitation, and this certainly leads to an increase in the yield of lycopene and β-carotene. At low ultrasonic intensities, the thermal effect can be ignored because the heat produced by the ultrasound is completely diffused. With the further increase in ultrasonic intensity, however, the cavitation effect becomes less important than the thermal effects during the extraction of sensitive products such as carotenoids [79,87]. Chen et al. [29] claimed that, during sonication, the extreme physical conditions of temperature and pressure caused the carotenoid isomerization. In addition to improving extraction efficiency, high ultrasonic power could cause thermal degradation to thermally sensitive components such as β-carotene [93,94]. As a result, the lycopene content increases over time from 15 min to 30 min in all ultrasonic power ranges. The β-carotene, on the other hand, begins to decline at 90 W. This can be explained by the differences in sensitivity of lycopene and β-carotene in thermal effects. In order to optimize lycopene extraction parameters by combining various techniques including sonication [95], Amiri-Rigi and coworkers studied the effect of the different ultrasonic intensities (30, 50, 70 and 90 W) and of the sonication durations (3, 5 and 8 min) on the lycopene content in the microemulsion phase. In a constant exposure time, the lycopene content in the microemulsion phase increased significantly as the ultrasonic power increased from 30 to 50 W. However, as the ultrasonic power increased beyond 50 W, the amount of lycopene extracted saw a slight decline. The increase in the ultrasound exposure time in constant power, on the other hand, resulted in a progressive reduction of the concentration of lycopene with the exception of the 30 W in which the extractability was improved by increasing the exposure time. Ultimately, the highest lycopene recovery was achieved using an ultrasonic power of 50 W and an exposure time of 3 min. Subsequently, the sonication power of 50 W was chosen for further investigation to see if a reduction in the exposure time to sonication was possible while improving the total recovery of the substance. From the reported data, the amount of lycopene extracted slightly decreased with an increasing sonication duration from 30 to 180 s, while no significant differences were shown at 30, 60, 90 and 120 s. Overall, the power of 50 W and the exposure time of 30 s were adopted as optimal operating parameters for the sonication pretreatment of tomato industrial waste powder. Studies also show that ultrasonic degradation of cellular tissue is extremely rapid and occurs within the first 30 s of sonication treatment [96]. Probably, the reduction in the concentration of lycopene at an increasing intensity of sonication may be due to the activity of enzymes, such as lipoxygenase, because of greater fragmentation of the cell wall induced by sonication. Lipoxygenase is able to oxidize lycopene and result in the isomerization of the double bonds of lycopene from *trans* to *cis*. In a previous study by the same authors on wet tomato pomace (mixture of skins and seeds), with a more intense sonication, a different treatment (power 20–37 W, temperature 10 °C, 15 min) was necessary to improve the efficiency [95].

## 6. Microwave-Assisted Extraction

Among the eco-friendly techniques, microwave-assisted extraction (MAE) has been accepted as a potential and powerful alternative for the extraction of bioactive compounds from plant material [97,98] and food industrial residues [99]. MAE combines microwave and traditional solvent extraction. In fact, during MAE, the solvent penetrates into the solid matrix and soluble products are extracted into a fluid until reaching a concentration limited by the characteristics of the solid. Microwaves are electromagnetic waves with frequencies ranging from 300 MHz to 300 GHz with two oscillating fields that are perpendicular such as electric field and magnetic field. These electromagnetic waves change the cell structure, a principal difference with respect to conventional extraction methods (solid–liquid or simply extraction). Whereas in conventional extraction the heat is transferred from the heating medium to the interior of the sample, in MAE, the heat is dissipated volumetrically inside the irradiated medium (volumetric heating) [100]. MAE becomes a suitable option in the place of traditional techniques thanks to its advantages: shorter extraction time, higher extraction rate and lower use of solvents and costs [101]. The first use of MAE goes back to 1986 by Ganzler et al. [102]. The authors reported the possibility to use MAE for extracting diverse types of compounds from soil, seeds, food and feed. Subsequently, several studies have reported the excellent performance in terms of recovery of the bioactive and nutritional components compared to other traditional extraction techniques. The basic principle of this extraction technique is that the moisture inside the cell evaporates due to the heat generated by the microwaves, and this process generates a high pressure on the cell wall causing modification of the physical properties of the biological tissues. This results in an increase in the porosity of the biological matrix which allows better penetration of the extraction solvent [103]. There are several factors that influence this technique: power, frequency and time of application, moisture content and size of the sample matrix plates, type and concentration of the solvent, ratio between solid and liquid, extraction temperature, extraction pressure and number of extraction cycles [104]. Among these factors, the choice of solvent is considered the most important critical point. Generally, the choice is made considering three physical parameters: (i) solubility, (ii) dielectric constant and (iii) dissipation factors. Solvents with a high dielectric constant, such as “water” and polar solvents, can absorb high microwave energy and are usually better solvents than non-polar ones [90,103]. In order to maximize the recovery of lycopene from tomato by-products, different auxiliary extraction technologies have been tested. Ho et al. investigated a possible alternative to conventional extraction methods using, as a matrix to be extracted, tomato peels derived from a local processing plant as a by-product in the production of tomato concentrate [105]. The skins were first neutralized with hydrochloric acid until a pH of 7 was obtained and then dried. In order to better facilitate extraction, the skins were ground until a particle size < 0.5 cm was achieved and then used. The response surface methodology (RSM) was used for obtaining the optimum conditions (solvent, time and microwave power) to recover lycopene and evaluate the effect of treatment on all-*trans* yields. The optimal conditions were: 0:10 solvent ratio at 400 W that provided 13.59 mg/100 g (all-*trans* lycopene). RSM indicated that ethyl acetate was a preferable MAE solvent to recover lycopene with respect to hexane (that extracted less lycopene). HPLC-DAD showed a clear improvement of all-*trans* and total lycopene yields by using MAE, with respect to conventional methods that led to higher amounts of *cis*-isomers [105]. This technique was also combined with ultrasound, creating ultrasound microwave-assisted extraction (UMAE). Lianfu et al. compared these two methods applied to lycopene extraction from tomatoes, with the aim of highlighting their advantages and disadvantages. The authors analyzed the effect of power (in UMAE), temperature (in UAE) and time and solid/solvent ratio (in both extraction techniques). In this work, RSM was used for obtaining the best conditions (solvent, time and microwave power) for lycopene extraction. The analyzed material was the tomato puree obtained after a centrifugation process. The optimum of UMAE was reached at: microwave power, 98 W; extracting time, 367 s; solvent/tomato paste ratio, 10.6:1. Differently, the optimum for UAE was: extracting temperature, 86.4° C; extracting time, 29.1 min; solvent/tomato paste ratio, 8.0:1. By comparing these two methods, it is evident that UMAE overcomes the gaps of UAE, being, therefore, a more attractive method [94].

## 7. Microemulsion Technique

The microemulsion technique (MET) represents another promising extraction method thanks to the exceptional physicochemical properties such as perfect stability, low viscosity, great solubilization capacity for both hydrophilic and lipophilic compounds and the increase in bioavailability of nutraceuticals. This technique, using a wide variety of surfactants, has already been successfully applied in the extraction of various nutraceuticals and organic compounds such as phenols, enzymes and proteins from liquids [106], oils [107,108] and proteins and glucosinolates from oilseed cruciferous meals [109]. This lycopene extraction was developed using a lecithin-based olive oil microemulsion [110] consisting in a water-based formulation with a small droplet size. In this study, soybean lecithin was used as an emulsifier, 1-propanol was used as a co-surfactant since it is considered safe in food preparation as a flavoring and coloring agent [111], while olive oil was used to enhance lecithin solubility. The optimum ratios of lecithin/1-propanol, olive oil and water (53.33:26.67:10:10 %wt) resulted in a very high extraction yield (88%) from tomato pomace after four extraction cycles. Other studies [96,112] investigated the use of different emulsifiers and co-surfactants in a multi-step extraction where a pretreatment with ultrasounds (50 W, 30 s) and pectinase (60 min, pH = 4.5, 45 °C) was applied before the microemulsion extraction of tomato waste provided the highest extraction yield when saponin and glycerol were used as a surfactant and co-surfactant, respectively.

## 8. Water-Induced Hydrocolloidal Complexation

This kind of extraction is based on the complexation of lycopene and pectin induced by the addition of water to tomato pomace followed by the separation of lycopene and pectins using solvent extraction. In the first step, a dispersion of colloidal particles from tomato pomace in a water solvent is formed presumably because pectins act as a natural emulsifier [113] dispersing the lycopene bodies released by tomato cells into the solvent. Two successive centrifugations at different rpm values allowed separating debris and colloidal complex; lastly, the recovery of lycopene and other carotenes from the complex can be performed by a small amount of organic solvent. By this extraction, optimized using response surface methodology, the maximum recovery obtained was of 9.43 mg carotenoid fractions/100 g tomato pomace, with a purity of carotenoid-rich fractions of 92% [114].

## 9. Conclusions

Food waste and by-product production, occurring during food processing, is becoming an ever-increasing environmental issue. With the aim of increasing the eco-sustainability of the food processing industry and reducing the impact on the environment, a promising strategy is to reuse and exploit by-products before they become waste. Many of these biomaterials are, in fact, a source of valuable compounds, having, therefore, powerful nutritional and functional use. Nonetheless, reusing components from by-products is still in its infancy, highlighting the need to invest in research and for new recovery technologies. Recently, numerous advances have been made in conventional and novel techniques successfully applied for recovery and reuse of waste from industrial tomato processing, in particular for extracting lycopene from TBPs. Indeed, COSE shows several drawbacks, mainly due to the toxicity of solvent traces that can remain in the extract and the high environmental impact, and, therefore, new and more eco-sustainable techniques have been investigated. On one hand, pretreatments of TBPs before COSE, including PEF and EAE, allowed reaching high extraction yields and shorter extraction times, but they still require organic solvents’ use downstream. On the other hand, various innovative techniques have been recently applied for the extraction of lycopene from TBPs, such as SFE. This environmentally friendly method showed several advantages such as low operating temperatures (that allow avoiding thermal degradation), high yields and selectivity and fast extraction times, and it is also free of organic solvents’ residues. Yet, SFE is characterized by high power consumption, very expensive and complex equipment and elevated operating pressures that could represent a risk for the operators. Another promising extracting technique is UAE, an eco-friendly method that allows high efficiency, low energy consumption and equipment costs, high extraction yields, short extraction time and that is basically harmless. However, limitations are related to difficulty in scaling up, possible degradation of thermolabile compounds, highly reactive free radical generation, low selectivity and limited extraction volumes. Similarly, the MAE method is characterized by similar disadvantages compared to UAE, even if the equipment costs are higher and the possibility of thermal degradation occurs more rarely. Nonetheless, this eco-friendly method allows reducing the amount of organic solvents and reaching short extraction times and high extraction yields. Two very recently described extraction methods are the microemulsion technique and the water-induced hydrocolloidal complexation, both promising and characterized by several advantages. Indeed, the first one allows high extraction yields, stability against oxidation and reduced use of organic solvents, even if it has some drawbacks (the residual presence of surfactants that could be risky for health and the development of complex systems that may be time-consuming). The water-induced hydrocolloidal complexation is characterized by high selectivity and high purity of the extracted compounds, but it needs a subsequent recovery step, and the extraction yields are comparable to that of classical organic solvent extraction. Taken together, these data could hopefully improve the awareness in reducing food waste as much as possible and increase the reuse of components from processing industry by-products.

## Figures and Tables

**Table 1:** Extraction methods for recovering lycopene from TBPs: advantages and disadvantages.

	Extraction Method	Main Features	Advantages	Disadvantages
	Classical organic solvent extraction (COSE)	solubilization of the components of interest into organic solvent/s added to the plant matrix	easily modifiable parameters (i.e., ratio solvent/matrix, temperature, extraction time) large production capacity easy continuous operation	long process time toxicity of solvents traces that can remain downstream purification operations high environmental impact high health risks (i.e., use of highly flammable and/or toxic solvents)
Pretreatments before COSE	Pulsed electric fields treatment	application of electric field pulsing on plant matrices that induces electropermeabilization	high extraction yields no thermal effect low operation cost short process time no isomerization or degradation	dependence on medium composition (conductivity) high cost of the equipment
	Enzyme-assisted extraction	use of enzymes catalyzing the hydrolytic cleavage of structural components of the cell wall of the waste product	high extraction yields reduction in extraction time reduction in using organic solvent high selectivity	high enzyme cost currently available enzymes cannot hydrolyze cell walls completely difficulty of enzyme recovery high dependence on pH, temperature, metal ions, etc.
	Supercritical fluid extraction	use of supercritical fluids as the extracting solvents to separate component/s from the matrix	eco-friendly method high purity low operating temperatures (no thermal degradation) free of organic solvents’ residues fast and high yield high selectivity	high power consumption very expensive and complex equipment elevated operating pressures consistency and reproducibility may vary in continuous production ^1^
	Ultrasonic-assisted extraction	the ultrasound waves cause a mechanical impact, allowing greater penetration of the solvent into the plant body (“sponge effect”)	eco-friendly method high efficiency low energy consumption low equipment cost high extraction yields reduction in extraction time harmless use of small amount of organic solvents	scale-up not feasible possible degradation of thermolabile compounds highly reactive free radical generation low selectivity limited extraction volumes lack of uniformity in the distribution of ultrasound energy
	Microwave-assisted extraction	microwaves heat solvents that contain samples, thereby partitioning analytes from the matrix into the solvent	eco-friendly method reduced use or no use of organic solvents possibility to avoid thermal degradation short extraction times high extraction yields	difficult to scale up potential risk of explosion in case of closed vessel operating conditions limited extraction volume high costs of microwave set-up limited choice of the extraction solvents
	Microemulsion technique	formation of thermodynamically stable dispersion of two immiscible liquids in the presence of surfactants (microemulsions) that improve the solubilization capacity of both liquids	high extraction yields ease and economical scale-up efficient at room temperature short extraction times reduced use or no use of organic solvents stability against oxidation	surfactants could be risky for health phase inversion microemulsions could require development of complex systems that may be time-consuming
	Water-induced hydrocolloidal complexation	formation of lycopene and pectin colloidal complexes in an aqueous environment that can be recovered by sedimentation or centrifugation	small amount of organic solvents high selectivity high purity	need of a subsequent recovery step extraction yield comparable to that of classical organic solvent extraction

^1^ see Reference [35].

## Data Availability

The data presented in this study are available on request from the corresponding author.

## Sample Availability

Samples of the compounds are not available from the authors.
